# Recurrence rate of oral squamous cell papilloma after excision with surgical scalpel or laser therapy: A retrospective cohort study

**DOI:** 10.4317/medoral.22943

**Published:** 2019-06-25

**Authors:** Jorge Toledano-Serrabona, Marta López-Ramírez, Alba Sánchez-Torres, Antoni España-Tost, Cosme Gay-Escoda

**Affiliations:** 1 DDS. Fellow of Master’s Degree of Oral Surgery and Implantology. School of Medicine and Health Sciences, University of Barcelona, Barcelona (Spain); 2 DDS, MS. Master’s Degree Program in Oral Surgery and Implantology, School of Medicine and Health Sciences, University of Barcelona (Spain); 3 DDS, MS. Associate professor of Oral Surgery. Master’s Degree Program in Oral Surgery and Implantology, School of Medicine and Health Sciences, University of Barcelona. Researcher at the IDIBELL Institute, Barcelona (Spain); 4 MD, DDS, MS, PhD. Associate professor of Oral Surgery. Master’s Degree Program in Oral Surgery and Implantology. Director of the Master of Laser Dentistry degree program, School of Medicine and Health Sciences, University of Barcelona. Coordinator of the European Master’s Degree in Oral Laser Applications (EMDOLA) degree program. Researcher at the IDIBELL Institute, Barcelona (Spain); 5 MD, DDS, MS, PhD, EBOS, OMFS. Chairman and Professor of Oral and Maxillofacial Surgery, School of Medicine and Health Sciences, Barcelona. Director of the Master’s Degree Program in Oral Surgery and Implantology (EFHRE International University/FUCSO). Coordinator/Researcher at the IDIBELL Institute. Head of the Oral Surgery, Implantology and Maxillofacial Surgery, Department of the Teknon Medical Center, Barcelona (Spain)

## Abstract

**Background:**

The aim was to describe the recurrence rates of Oral Squamous Cell Papilloma (OSCP) following surgical treatment with surgical scalpel and two different lasers (CO2 or Er,Cr;YSGG) and to determine the clinical and histopathologic features of these lesions.

**Material and Methods:**

A retrospective cohort study covering a period of 12 years (1997-2009) that included patients diagnosed of OSCP treated with surgical excision was performed. Data was processed using SPSS 22.0 (SPPS Inc. Chicago, USA) and a descriptive and bivariate analysis were conducted.

**Results:**

A total of 37 histopathologically confirmed OSCP in 36 patients, 19 women (52.7%) and 17 men (47.2%) with an average age of 33.4 years (14-86 years) were included. Twenty-two cases were treated by excision with surgical scalpel excision, 11 with CO2 laser and 3 with Er,Cr:YSGG laser. The mean age was 35.4 years (14-86 years) and the distribution by gender was 19 women (52.7%) and 17 men (47.2%). The most common locations were the palate in 14 cases (37.8%), followed by the tongue in 11 cases (29.7%) and gingiva with 5 cases (13.5%). The average size of the lesions was 4.25 mm in diameter, with a mean evolution time of 5.9 months. The recurrence rate was slightly higher with the CO2 laser (14.3 %) in comparison with the conventional scalpel (10%). No recurrences for Er,Cr:YSGG were found.

**Conclusions:**

No differences for recurrence rates for OSCP between groups were found. The recurrence rate is low, happening usually before 15 months of follow-up. OSCPs are lesions usually appearing in patients between 30 and 50 years of both genders and located predominantly on the palate, tongue and gingiva.

** Key words:**Oral squamous cell papilloma, squamous cell papilloma, CO2 laser, recurrence.

## Introduction

According to the current classification of the World Health Organization, Oral Squamous Cell Papilloma (OSCP) is a benign hyperplastic exophytic localized proliferation with a verrucous or cauliflower-like morphology, which its base may be sessile or pedunculated (1). The color of the lesion may vary from white to pink, depending on the level of keratinization and vascularization (2). It usually appears as a single lesion that grows rapidly in a period of few months to a maximum of 1 cm in diameter (3). The most common sites are the soft palate, lips, tongue and gingiva, although any area of the oral cavity can be affected (1,2,4). Generally, the OSCP occurs in patients aged between 30 and 50, although it can appear at any age. There is no consensus about the predilection for gender (1).

Histologically finger-like projections of fibrovascular tissue covered by hyperplastic and hyperkeratotic stratified squamous epithelium and marked granular cell layer are observed. Koilocytes (keratinocytes with pyknotic nuclei, perinuclear space and cytoplasm condensed) are usually seen in lesions of short time of evolution. At the base small lymphocytic inflammation foci can be observed, which are often poor unless the lesion is subjected to repeated trauma or other irritations (1-3).

The differential diagnosis is established with the following entities: verruca vulgaris, multifocal epithelial hyperplasia, condyloma acuminatum, and papillary squamous cell carcinoma (1,5).

The treatment of choice is the complete surgical excision including the base of the lesion and a small area of surrounding tissue to prevent recurrence (1,3). Apart from the surgical scalpel, the use of laser has also been proposed to eliminate OSCP (6).

The main objective of this study was to determine the recurrence rate of OSCP after surgical excision with the surgical scalpel or laser (CO2 and Er,Cr:YSGG). The secondary objective was to describe the clinicopathological features of these lesions.

## Material and Methods

A retrospective cohort study over a period of 12 years (1997-2009) including patients with a definitive diagnosis of OSCP by a histopathological exam and treated by surgical excision at the Oral Surgery Department of the University of Barcelona study was carried out. This study was performed according to the Strengthening the Reporting of Observational studies in Epidemiology (STROBE) Statement (7).

The following variables were registered: age, gender, race, smoking habit, presence of a traumatic factor, location and clinical appearance of the lesion (size, color and surface features), time of evolution until the histopathological diagnosis, symptoms, excision modality, and recurrence. Patients were scheduled to attend a follow-up appointment.

OSCPs were excised under perilesional local anesthesia with 4% Articaine with 1:100.000 epinephrine (Ultracain; Normon, Madrid, Spain), except the cases removed with the Er,Cr:YSGG laser, in which no local anesthetic was administered. In all cases an excisional biopsy was performed with peripheral and in-depth safety margins.

**Figure 1:**
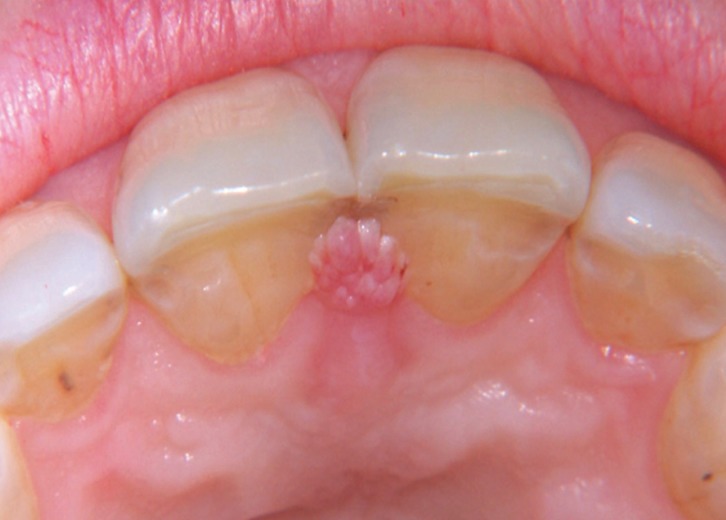
Preoperative view of OSCP located in the interincisal palatine papilla.

**Figure 2:**
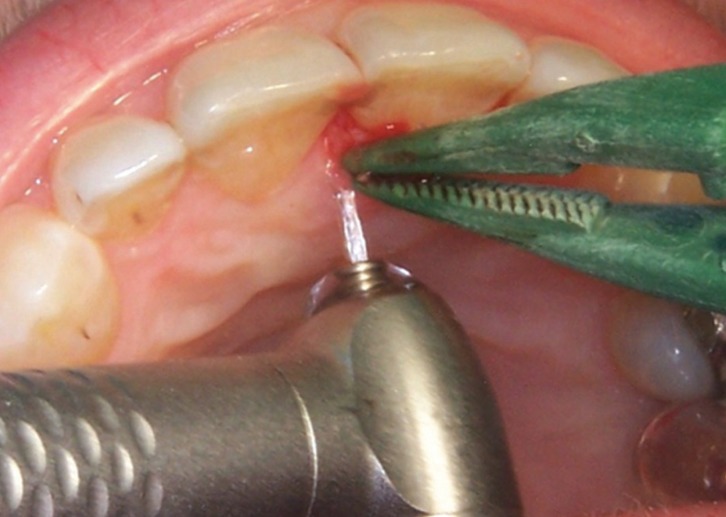
Excision of the lesion with the Er,Cr:YSGG laser.

The CO2 laser (Lasersat 20W. Sharplan 1020, Tel Aviv, Israel) was used with a power between 3 and 5 W. The effect of cutting was achieved using a spot of 0.8 mm of diameter, and a focused laser to 3 W of power at a continuous mode (power density of 600 W/cm2), and the coagulation effect was obtained using a spot of 1.5 mm of diameter and a power of 5 W at continuous mode (power density of 280 W/cm2). The Er,Cr:YSGG laser (WaterlaseTM, Biolase Technology, San Clemente, USA.) was used at a power of 1.5 W, frequency 20 Hz, a pulse energy of 75 mJ, 0.8 mm of spot diameter and 15.0 J/cm2 of energy density per pulse (fluence) under constant refrigeration (7% air and 11% water). The laser impacts were directed at the base of the lesion, surrounding and in direct contact with the tissues (Figs. 1,2). Once the elimination was completed, the control of hemostasis was carried out. The wounds after the use of surgical scalpel were sutured whereas those caused by the laser were healed by secondary intention. The samples were immersed in a solution of 10% formalin and sent to perform the histopathological study.

A postoperative treatment was prescribed for cases operated with surgical scalpel consisting in 0.2% chlorhexidine gel (Laboratorios Lacer, Cerdanyola del Vallés, Spain) application on the surgical area in cases operated with scalpel. In addition, a pharmacological oral treatment was prescribed, ibuprofen 600 mg (Kern Pharma SL, Terrassa, Spain) every 8 hours for 4 days and paracetamol 1g (Cinfa, Huarte, Spain) as rescue medication. No medication was prescribed for the patients treated by laser.

All data was processed using SPSS 22.0 (SPPS Inc. Chicago, USA). A descriptive and bivariate analysis by means of a Chi-square test in order to assess differences in recurrence rates between groups were performed.

## Results

Out of 2.638 biopsies carried out during the period from January 1997 to October 2009, 37 histopathologically confirmed OSCP in 36 patients were found. In our Oral Surgery Department, OSCP had an incidence of 1.4%. Twenty-two patients were treated by excision with surgical scalpel, 11 with CO2 laser and 3 with Er,Cr:YSGG laser. The mean age was 35.4 years (14-86 years) with a peak incidence between 30-50 years and the distribution by gender was 19 women (52.7%) and 17 men (47.2%). Thirty-five patients were Caucasian and 1 was Asian. Seven patients were heavy smokers (> 20 cigarettes/day) and 4 patients were light smokers (< 10 cigarettes/day). Regarding the existence of a traumatic or irritant factor, 7 patients (19.4%) showed a poor fit of their removable partial denture and 4 patients (11.1%) reported a continuous biting habit in their tongue.

In 32 patients (89%) the lesion was unique, but 3 cases showed multiple forms with small confluent papillomatous lesions. In one of these patients, the lesions were observed in two different locations, one on the floor of the mouth and another in the hard palate.

Twenty-four lesions (64.9%) were discovered by the own patient either by direct visualization or by the appearance of nonspecific oral discomfort. The rest of the lesions (35.1%) were detected by the dentist at a routine examination.

Twenty-nine OSCP (78.4%) were white and 8 (21.6%) had a pink color. Although most lesions 33 OSCP (89.2%) showed a typically cauliflower surface, 4 cases (10.8%) had a smooth surface.

**Figure 3:**
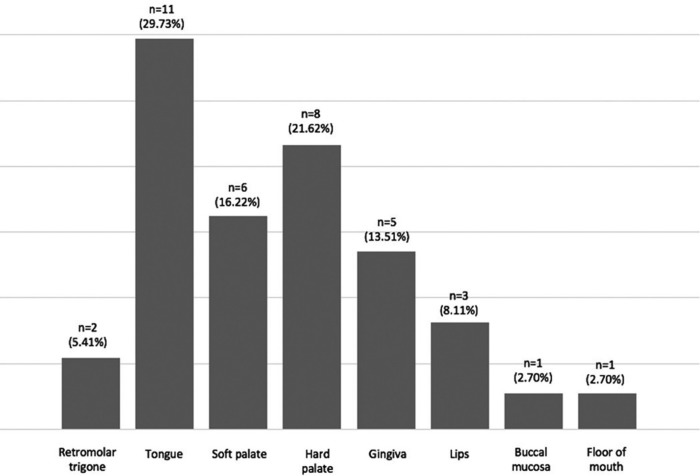
Distribution of OSCP according to location.

Figure 3 shows the location for the lesions, being the palate the most frequent area of apparition (14 cases, 37.8%). The average size of the lesions was 4.25 mm in diameter (ranging from 2 to 10 mm), with a mean evolution time of 5.9 months (ranging from 1 to 31 months).

Two out of the 36 participants did not attend the follow-up appointment; so, recurrences were only assessed in 34 patients. A total of 4 recurrences, 3 in men (1 smoker) and 1 in a woman (poorly fitting removable partial denture) were observed. The lesions appeared in the same location as the initial lesion, two cases in the lower lip, one in the retromolar trigone and one in the gingiva, with an average size of 4.8 mm. From these, 2 lesions from 22 (9%) had been excised by the surgical scalpel and 2 from 11 had been operated by the CO2 laser. The recurrence rate was slightly higher with the CO2 laser (18.2%) in comparison with the conventional scalpel (10%). No recurrences for Er,Cr:YSGG laser were found. All recurrences occurred between 4 and 15 months after excision and were again removed with the same treatment that had been initially submitted. One of the OSCP removed with scalpel recurred up to 2 times. No differences were found for recurrence rates between three groups (*p*=0.928).

## Discussion

The incidence of 1.4% for OSCP in our Oral Surgery Department was slightly lower than the 2.4% reported by Abbey *et al.* (4) but similar to 1.9% obtained by Bhaskar (8) in a sample of 20.575 biopsies. In the present study the age of occurrence and gender predilection agree with literature (1,9).

The appearance of OSCP is associated with HPV infection. In 1967, Frithiof and Wersäll (10) demonstrated the presence of viral particles very similar to HPV in oral papillomas. It was not until 1982, when Jenson *et al.* (11) reported about the antigenic structure of HPV by immunohistochemical methods. Since then, more precise and sensitive techniques for detection of HPV as the hybridization of nucleic acids and the chain reaction (PCR) had been developed. This technique allowed to detect the DNA sequence of HPV 6 and 11 in a 50% to 68% of OSCP (12).

The last 20 years there has been a growing interest in detection of HPV by their relationship in the pathogenesis of squamous cell carcinoma of the oral cavity (13,14).

Although the major risk factors for head and neck cancer remains the tobacco and alcohol, HPV was recently responsible of malignant lesions in oropharyngeal cancer (13).

The OSCP is generally asymptomatic. This reason can explain the 5.9 months of evolution found in our study until patients turn back the facultative. In a study published by Abbey *et al.* (4) more than 50% of the lesions had remained between 2 and 11 months, and over 30% between 1 and 10 years until its removal.

The most prevalent site in our series was the palate (37.84%), followed by the tongue (29.73%). Interestingly we only detected a case of OSCP at the level of the uvula, being this location described by some authors as one of the most frequent locations (4). These results may suggest that the palate and tongue are two of the locations of the oral cavity more susceptible to microtraumas and irritations.

HPV can be spread by direct cutaneous, oral or sexual (vaginal or anal) contact with an infected person and there is also the possibility of vertical transmission from mother to child during delivery. Promiscuity, age of onset sexual activity, oral sex practice, smoking and immunosuppression are the main risk factors for HPV infection and progression to an oropharyngeal cancer. Most sexually transmitted infections are caused by HPV types of high risk, with HPV16 being the most frequent (13,14). As persistent infection with HPV is associated with cervical cancer (15), it would be advisable that the partners of patients with OSCP did routine check-ups with the dentist and the gynaecologist for possible papillary lesions in both regions.

The treatment of choice for OSCP is surgical excision either with a surgical scalpel or with other treatment options such as laser surgical excision (6) exist although a small number of publications can be found in the literature.

Since the introduction of laser in early 70’s, the use of CO2 laser has been useful in a variety of surgical procedures such as removal of soft tissue lesions of the oral cavity (16). There are other surgical lasers that can be used for the same purpose such as, Nd:YAG, Nd:YAP, diode, Ho:YAG and Er:YAG.

White *et al.* (16) made the removal of 64 benign soft tissue, among which there was two cases of OSCP that were removed with the CO2 laser, but both relapsed within a few months. Comparing these results with the OSCP treated with a surgical scalpel, we can see that the recurrence rate is lower (4.1%) with this technique (4).

Regarding the use of Er,Cr:YSGG laser we just find published one case report of a pediatric patient which underwent the excision of a OSCP located in the distal gingiva of the upper left first permanent molar. At 6 months follow up no evidence of any recurrence (6).

It is considered that a complete excision of the lesion must be performed in order to prevent recurrence (3,9), so the probable origin of our recurrences could be the incomplete removal of the lesion, leaving residual pathological tissue.

Unfortunately, our study was too small to permit a robust analysis of recurrence rates in patients who underwent different excision modalities, because only 37 OSCPs were diagnosed.

## Conclusions

No differences for recurrence rates for OSCP between groups were found. The recurrence rate is low, happening usually before 15 months of follow-up. OSCPs are lesions usually appearing in patients between 30 and 50 years of both genders and located predominantly on the palate, tongue and gingiva.
